# The effect of low-fidelity simulation training on breastfeeding knowledge, practice, and self-efficacy among young lactating mothers in Tanzania: A quasi-experimental study

**DOI:** 10.1371/journal.pone.0285392

**Published:** 2023-11-28

**Authors:** Rogers Kaiza, Angelina A. Joho

**Affiliations:** Department of Clinical Nursing, School of Nursing and Public Health, The University of Dodoma, Dodoma, Tanzania; National Defence University - Kenya, KENYA

## Abstract

**Background:**

Exclusive breastfeeding (EBF) is of paramount importance for the survival, growth, and development of neonates. Lack of EBF puts mothers and their babies at high risk of many complications. Mothers may end up having breast engorgement, cracked nipple, mastitis, breast pain, and backache. Babies may acquire postnatal HIV transmission, reduce weight, stunting, poor cognitive and motor development, and increase the risk of diarrhea disease and respiratory infection. Breastfeeding training has been provided immediately after a women’s birth. However, young mothers are still staggering with breastfeeding.

**Research aim:**

We aim to assess the effect of low-fidelity simulation training on breastfeeding knowledge, practice, and self-efficacy among young lactating mothers and we will also assess the impact of simulation on infants’ weight in Tanzania.

**Methods:**

The study will be a health facility-based quasi-experimental design. The study will have four phases: baseline survey, intervention, immediate assessment, and two-month end-line follow-up. The assessment will focus on participants’ breastfeeding knowledge, practice, and self-efficacy. Furthermore, infant weight will also be assessed during baseline and end-line. A total of 261 young lactating mothers who have first baby with their infants aged 0 to 2 months will be included, whereby 87 young lactating mothers will be in an interventional group and 174 will be in the control group. The intervention will have four packages: 1) group lecture education and interactive on the importance of breastfeeding 2) videos, 3) pictures, and 4) simulation in the umbrella LVPS using the wearable Lactation Simulation Model (LSM) and newborn manikins (Global Health Media and Laerdal baby). At the end of the study, all groups will be given education brochures which will be in the Swahili language for easy understanding. Data will be analyzed using SPSS version 23. An independent T-test and repeated measures ANOVA will be used in this study to compare the difference between the mean of the 2 groups.

**Discussion:**

This study aims to generate evidence of the effect of simulation on improving breastfeeding knowledge, practice, and self-efficacy. We expect the study findings to inform the stakeholders and policymakers on formulating breastfeeding education and simulation training that will improve women’s breastfeeding knowledge, practice, and self-efficacy and improve infant health.

## Background

The World Health Organization (WHO, 2017) [1] describes breastfeeding as the cornerstone of child survival, nutrition, and development. It is recommended to breastfeed infants under six months exclusively with only breast milk without introducing any other food [2]. Effective breastfeeding is paramount to a baby’s survival, as infants who are not well breastfed have a 14 times higher risk of dying within 6–11 months of their life than breastfed infants [3–5].

Exclusive breastfeeding requires an effective sucking technique to ensure milk transfer and to prevent breastfeeding complications [3, 6]. Furthermore, effective breastfeeding can be achieved when mother and baby are well-positioned and the baby is well attached to the mother’s breast. This will facilitate effective breastfeeding, and increase the production and excretion of milk. In addition, effective breastfeeding may reduce backache, breast pain, breast engorgement, cracked nipple, mastitis and risk of postnatal HIV acquisition [3, 6, 7]. Also, reduce early discontinuation breastfeeding [8]. On the other hand non-exclusive breastfeeding may lead to low intake of breast milk, poor weight gain, stunting, low immunity [9] (Tiruye, Mesfin, Geda, & Shiferaw, 2018), increased diarrhea diseases, and respiratory infection [10].

Factors associated with non-exclusive breastfeeding include inadequate knowledge about breastfeeding, low education level, working outside home [11, 12], inadequate ANC visits and poor income [13]. In additional, various studies provide evidence of poor breastfeeding techniques and inadequate knowledge of breastfeeding practice. Often parents have inadequate knowledge about exclusive breast feeding [14].

Inadequate breastfeeding knowledge and practice are thought to be as a result of the traditional teaching method which is facilitator and content-focused [15]. Studies have reported shortcomings of using the lecture method of teaching such as being easily forgotten, participants may lose interest within a short time, not being effective for the acquisition of psychomotor skills, does not provide effective feedback on learners’ performance [15]. Various pedagogical technologies with interactive simulation-based training have been developed to overcome these challenges and range from low to high fidelity depending on the ability to simulate the reality continuum [16]. The low-fidelity simulation-based training has the constructs of hands-on activities that resemble the real clinical environment using simple mannequins which have a minimal physiological response that provides the expected learning outcome [17].

Simulation involves imitating the behavior of a real situation or process and learners respond to the situation as is it done in real conditions under natural circumstances [18]. It is an artificially pre-planned condition with the use of instruments or manikins as a representation of real condition in real life. Learners transforming knowledge into practice to develop skills and when learner encounters a similar situation can apply the learned practice to make a decision and solve the problem [19] Webber et al [20] reported that, learners who had participated in simulation training were able to position themselves and attach their infants to the breast very well and confidently. Moreover, the trained learners were able to instruct other lactating mothers confidently on the same. The study by Sabrian et al [21] suggested simulation can be applied as educational support during breastfeeding counseling as it enables learners to practice the learned skill with actual skills, as it first be done with non-human objects such as wearable breasts and baby dolls, even with mistakes, it allows several repetitions to be collected, it helps to develop competence and retain the memory of the learner.

The National Road Map Strategic Plan target of increasing exclusive breastfeeding was 90% by 2015 [22, 23]. However, this strategic plan has not been achieved as studies conducted in Tanzania reported that EBF is still suboptimal. A study conducted in Zanzibar reported the prevalence of EBF was 20.8% in 2015 [24], in Muheza was 24.1% in 2016 [25], in Coastal Region of Tanzania was 30% in 2016 [26].

Despite several adoption and initiatives made by the Tanzanian government and stakeholders to improve EBF, Dodoma Region has 47.7% level of EBF [26]. This is lower than the national coverage of 59% [26], and certainly lower than the WHO recommendation of 90% [27]. Therefore, we aim to [1] assess the effect of low-fidelity simulation training on breastfeeding knowledge, practice, and self-efficacy of young lactating mothers in the Dodoma Region and (2) assess the impact of simulation on infants’ weight.

### Research objectives

The objective of this study is to assess the effect of low-fidelity simulation training on infant weight, knowledge, practice, and self-efficacy of young lactating mothers on breastfeeding techniques in the Dodoma region.

## Methods

### Study site

The study will be conducted in Dodoma Region. Dodoma Region is found in the central zone of Tanzania. It is bordered by four regions namely Manyara in the North, Morogoro in the East, Iringa in the South, and Singida region to the West. Much of the region is a plateau rising gradually from some 830 meters in Bahi Swamps to 2000 meters above sea level, in the highlands north of Kondoa. The region is divided into seven districts; Dodoma Urban, Chamwino, Bahi, Kongwa, Chemba, Kondoa, and Mpwapwa. According to the National Bureau of Statistics’ 2022 National Census, the population of Dodoma as 3,085,625, whereby males were 1,512,760 and females were 1,572,865 [28]. The region has 7 hospitals, 21 health centers, and 240 dispensaries. Tanzania National Bureau of Statistics in 2012 reported Dodoma has a total fertility rate of 5.5 [29]. Unpublished regional reports indicate that, neonatal deliveries in 2021 by district included; 18943 births in Dodoma Urban District, 12472 births in Chamwino District, 10289 births Kongwa District, 9663 births in Mpwapwa District, 6596 births in Bahi District, 6596 in Chemba District and 4330 in Kondoa District. The Ministry of Health reported that, Dodoma Region has a low rate (47.7%) of exclusive breastfeeding compared to the neighboring regions of Singida and Iringa 57.7% and 66.2%, respectively [26].

### Study design

A quasi-experimental study, with comparison and intervention groups, will be employed. The study will be divided into four phases: baseline survey, intervention, immediate assessment, and two-month end-line follow-up. The assessment will focus on participants’ breastfeeding knowledge, practice (positioning and attachment), and self-efficacy. Furthermore, infant weight will also be assessed during baseline and end-line.

### Phase one: Baseline assessment of outcome

The first phase will be a pre-assessment of the baseline data from both intervention and comparison groups on knowledge, practice, and self-efficacy related to the breastfeeding technique. The Baby’s weight will also be collected during the survey period. Data collection will be collected by the principal investigator and two research assistants recognized as National BEmCO nurse-midwives. This phase will involve the collection of baseline parameters 1) knowledge related to the importance of breastfeeding and the effect of not breastfeeding, 2) breastfeeding practice (positioning and attachment), 3) self-efficacy of mothers in breastfeeding, and 4) weight of infants. The baseline data will be collected over two weeks as was done by Crofts et al., [30]. Every health center will be treated as one group, to allow the researcher to be able to test for any variation among breastfeeding mothers within the health center [15]. The interviewer-administered questionnaire will be used for data collection. A questionnaire for assessing knowledge regarding breastfeeding benefits for both mothers and their infants will include knowledge of the mothers regarding baby’s attachment and position, the mothers’ position, and post-feeding position, as reported from a previous study conducted in India [31]. Questionnaire regarding maternal–related characteristics like age, level of education, previous breastfeeding experiences, sources of breastfeeding information and teaching environment, place of delivery, parity, and previous exposure to antenatal breastfeeding education. The questionnaire will be adopted from a study conducted in Ethiopia [9].

Concerning the assessment of breastfeeding practice which includes correct attachment, position and effective sucking of an infant during breastfeeding, a checklist named WHO/UNICEF B-R-E-A-S-T feed observation form of 1994, as was previously reported by Schubiger et al., [32] will be used. The checklist will have 13 items, that will be used to assess positioning and attachment, 7 items will be used for assessing the position, 4 items will be used for assessing attachment and 2 items will be used for assessing the effectiveness of sucking [32].

### Phase two: Intervention lecture, video, picture and education simulation (LVPS-Program)

This phase will be done for four weeks for the interventional group while the control group will receive lectures alone. The intervention group will have four packages: A lecture on the importance of breastfeeding, videos, picture, and education simulation training on attachment and position of an infant during breastfeeding by using the LSM and newborn manikins (Global Health Media and Laerdal baby).

### Lecture

Group session lecture will be facilitated by the licensed nurse-midwife and a recognized National BEmCO Nurse Midwife-Master Trainer (MTs) on breastfeeding techniques. The lecture method of training is the standard way that have been given to mothers in the health facilities. The venue will be one room within the reproductive and child health (RCH) clinic. The group size will be 6–8 mothers for easy understanding, we will use power point presentation as lecture delivery method, which will be delivered once and it will take 30-45minutes. The content of the lecture will cover the following; the importance of breastfeeding to the mother and her infant (cost-effective, easy to access, clean, safe, and containing antibodies that help in protecting the infant from childhood diseases such as diarrhea, pneumonia, also it reduces the risk of later obesity or cardiometabolic disorders to the infant, for mothers, women who exercise breastfeeding have reduced risk of breast and ovarian cancer, breastfeeding acts as a family planning methods), the effects of non-adherence of breastfeeding technique which include clicked and pain nipple, insufficient baby feeding, baby excessive crying, poor weight gain, poor intellectual [33]. At the end of lecture mothers will be allowed to ask questions. Facilitator (MT) will provide answers and clarification. Additionally, we will have printed pictures in case of power break down

### Video and pictures

After the lecture session, participants will be exposed to the video which will be adopted from Global Health Media videos in Swahili Language. Participants will watch the video in a group and it is expected that the video will take 15–20 minutes. A laptop and projector will be used for showing video in a selected room within the RCH clinic. The principal investigator will be together with mothers at the time of watching the video to monitor the level of attention paid by the participant. The video will show how to position and attach the baby to the breast for good production of milk. It will show the baby’s mouth is wide open, the chin is touching the breast, the baby sustains a rhythmic ’suckle/swallow/breathe’ pattern with periodic pauses, swallowing is audible, and the arm and hands are relaxed [34]. Also, we will show lactating mothers pictures showing the position and attachment of the baby during breastfeeding. These pictures will be adopted from WHO 2018.

### Simulation-based training (SBT)

The package of SBT will include baby’s positioning and attaching to the breast and also the mother’s position during breastfeeding. Group SBT in each health center will be provided for 30 minutes [15]. The SBT will be conducted in small groups of four to six lactating mothers per facilitator, in all health centers, with a participant-facilitator ratio of 1:6 [35]. SBT will be implemented using low-fidelity mannequins (wearable Lactation Simulation Model (LSM) and NeoNatalie^TM^). The validity of simulation-based training on breastfeeding techniques was previously established [36]. The simulation will be facilitated by MTs using LSM and newborn manikins. The breastfeeding manikins will be left in the health facility after training for healthcare providers to continue training breastfeeding techniques to mothers. The simulation will aim to empower mothers with breastfeeding techniques by showing mothers how to position and attach the baby to the breast and also showing the mothers how to position themselves during breastfeeding. The technique of breastfeeding will be adopted from WHO, 2016.

### Immediate assessment

The third phase is aimed at determining the acquisition of knowledge, practice, and self-efficacy during the training of lactating mothers regarding the importance of breastfeeding, and the effect of not adhering to breastfeeding [36]. Immediately after an intervention (LVPS) individual lactating mothers will be assessed for their understanding of the importance of breastfeeding, position, and attachment. Knowledge, practice, and self-efficacy of lactating mothers in breastfeeding will be assessed by using the same questionnaire and checklist which were used during the baseline phase.

### Phase four: Post-training assessment

**Fig 1:**
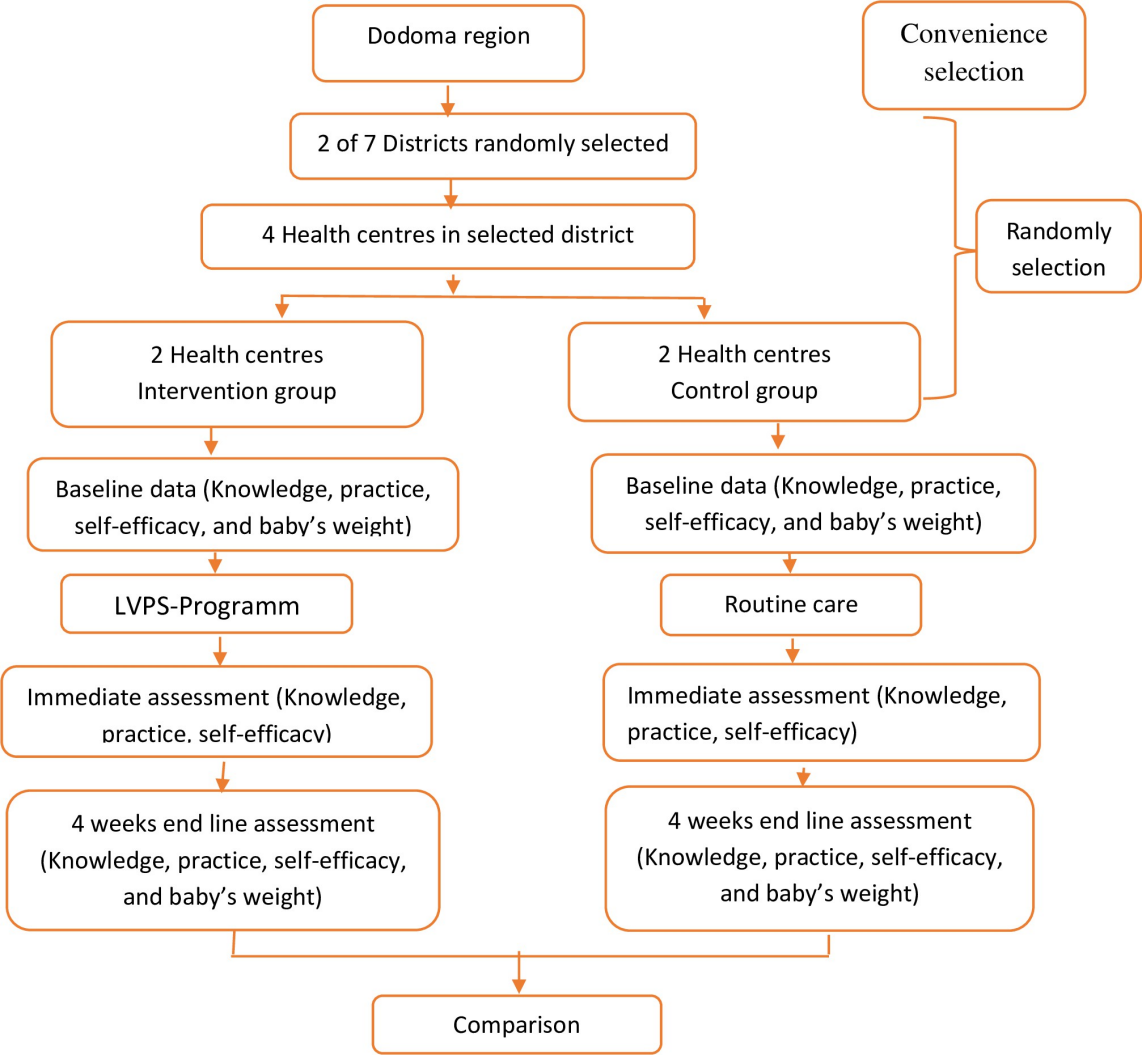
Flow chart of study design.

This will be a one-month post-training assessment of the knowledge, practice, and self-efficacy of breastfeeding mothers [34]. We also aimed to assess the weight of infants will be assessed. The MTs (recognized National BEmCO nurse midwives) trained in data collection during pre-training and post-training to maintain a consistency of information will be used. This phase will take four weeks, making the total study duration from the baseline to post-training assessment to be twelve weeks (Fig 1). The tools used in all phases will be the same.

### Study population

The study will involve lactating mothers for the first-time having babies aged 0–2 months, living in the Dodoma region attending reproductive and child health clinics from the selected health facilities. Lactating mothers with sick babies, babies with congenital malformations, premature babies, and lactating mothers who are living outside the catchment area will not be included in the study.

### Sample size

The sample size will be calculated by using a two-sample proportion using the formula below [37].


$$n=\frac{{\left\{Z\alpha \surd [\pi o(1-\pi o)]+2\beta \surd [\pi 1(1-\pi 1)]\right\}}^{2}}{{\left(\pi 1-\pi o\right)}^{2}}$$

Where n = maximum sample size, Zα = Standard normal deviation (1.96) at a 95% confidence level for this study, 2β = standard normal deviate (0.8) with a power of demonstrating a statistically significant difference before and after the intervention between the two groups at 80%, πo = Proportion of lactating mother at baseline skills on breastfeeding technique 34.1% [34], π1 = proportion of lactating mothers after intervention skills on breastfeeding technique 49.9 [34], After adjusting for 10% attrition, the sample size will be 87. The ratio of 1:2 will be used; 1 for the intervention group and 2 for the control group. For that case, 87 participants will be for the interventional group and 174 participants will be for the comparison group. Therefore, a total sample size of 261 young lactating women will be included in the study.

### Sampling technique

The convenience sampling technique will be used in selecting the Dodoma region, since has a low rate of exclusive breastfeeding of 47.7%, compared to the surrounding region Singida and Iringa 57.7% and 66.2% respectively [26]. Two districts in the Dodoma region will be selected randomly to be included in the study. There were pieces of paper that will be written YES or NO, the word YES will be used to represent the targeted district, and NO will be used to represent the district that will not be going to be included in the study. The procedure of drawing papers from the box by each research assistant will be used. Once the piece of paper has been chosen, it will not be included in the sample again and each research assistant will be allowed to **pick** only once. One district will be in the intervention group and the other in the control group. In each selected district, two health centers will be selected to be included in the study using simple random sampling. A proportionate stratified sampling method will be used to allocate the number of lactating mothers in each selected health center. Lactating mothers to be included in this study will be selected by using a systematic sampling technique.

### Variables measurement

The questionnaire will have five parts. Part I-sociodemographic characteristics, part II-knowledge, part III-self-efficacy, part IV-weight of the baby and part V practice. Knowledge will be measured using scores; the mean score will be considered to be the cut-off point for those with adequate and inadequate knowledge. Whereas, participants with less than the mean score will be considered to have inadequate knowledge and vice versa. Knowledge regarding breastfeeding techniques will have 12 items which will have four possible responses. However, at the time of data analysis, we will convert them into two options “Yes” for correct and “No” for incorrect responses. Young lactating mothers who will score correct 8 and more questions will be regarded to have adequate knowledge [31].

Breastfeeding Self-Efficacy will be assessed using a 14-item self-report tool, based on Bandura’s self-efficacy theory. The items contain 5- point Likert scales, whereas 1 indicating ‘not at all confident and 5 indicating ‘always confident’. The items are presented positively and they will be distributed ranging from 14 to 70 with higher scores indicating high breastfeeding confidence [38].

Regarding the practice of breastfeeding technique, assessment will be done using the WHO B-R-E-A-S-T feed observation checklist with criteria scores and grading of positioning and attachment. The position has a total score of seven criteria, whereby each criterion has a score of 1. Positioning will be graded as poor, average, and good based on the total scores of the criteria on the position of mother and baby. Scores of 1–2, will be graded as poor positioning, one criterion will be scored from the mother position and one criterion from the infant position. A score of 3–4, will be graded as average positioning if at least a score of one criterion from the mother position and two or three criteria from the infant position. A score of 5–7, will be graded as good if at least two criteria are scored from the mother position and three or four criteria from the infant position or if all criteria are scored from both the mother and infant position.

The correct attachment has four criteria that will be used for scoring and grading. The criteria are chin touching the breast, mouth wide opened, lower lip turned outward and more areola is seen above the baby’s mouth. The attachment has a total of four criteria, whereby each criterion has a score of 1. A score of 1, from any of the four criteria, will be graded as a poor attachment. A score of 2, from the four criteria, will be graded as an average attachment. A score of 3–4, from the four criteria, will be graded as a good attachment as reported in the previous study [39].

Effective sucking and milk transfer will be measured by asking mothers two questions; When initiating breastfeeding, does the sucking rhythm change to deep rhythmical sucks? 2) Is swallowing audible? [40, 41]. The sucking and milk transfer will have two possible responses “Yes” for correct and “No” for incorrect responses. Lactating mothers who will score correct 2 questions will be regarded to have their babies sucking effectively. Those who will score 1 or less will be regarded to have babies sucking ineffectively [40].

A weight measurement of an infant will be taken using a spring type infant weighing scale 1kg-1000g with trouser mechanical hanging baby scale. Infants will be measured weight while naked. We will also calibrate the accuracy of the weighing scale every time before weighing the infants by adjusting for zero error. The age of an infant will be determined by birth date which will be extracted from the immunization card. The WHO standards of age-gender specific z scores will be used for the measurement of weight according to age. Whereas defining Z-scores as a measure of standard deviations of the distance from the median value, adjusted for gender and age [41].

### Validity and reliability

To ensure the validity of the data the following will be taken into consideration. Tools will be shared with experts to evaluate the tools to suit the local environment (National BEmCO nurse-midwives, obstetrics and gynecology specialists, and pediatric medicine). Also, a pilot face validity will be done using 10% of the total sample size for the accuracy of the tools. We will also calibrate the accuracy of the weighing scale every time before weighing infants by adjusting for zero error. We will calculate Cronbach’s alpha coefficient for internal consistency measures to establish the levels of knowledge, practice, and self-efficacy. The questionnaire will be interpreted in Kiswahili for easy understanding.

### Statistical analysis

Statistical analysis will be performed using Statistical Package for Social Science (SPSS) version 23. Descriptive statistics will be used to analyze the sociodemographic characteristics and the results will be presented in proportions. An independent T-test and repeated measures ANOVA will be used in this study to compare the difference between the mean of the 2 groups. p < 0.05 will be considered significant.

## Discussion

The present study protocol covers design, outcome measures, sample size calculations, and procedures done in the intervention using a lecture on the importance of breastfeeding, videos, picture, and simulation training on attachment and position of an infant during breastfeeding by using the LSM and newborn manikins. The findings of this study will contribute evidence to the limited body of knowledge regarding simulation training, interactive teaching and education, use of video and pictures on improving breastfeeding knowledge, practice, and self-efficacy among young lactating mothers.

A previous study showed that simulation-based training is the appropriate method for improving breastfeeding knowledge, practice, and self-efficacy when it is used during breastfeeding training [21]. This study is expected to improve the knowledge, skill and confidence of lactating mothers in breastfeeding practice and thus improve the duration of exclusive breastfeeding and improve infant health. Also, the results can guide government and stakeholders on the improvement of policies and interactive educational methods for breastfeeding education.

### Strengths and limitations of the study

Having a follow-up period after intervention renders a design vulnerable to all internal validity threats because it is difficult to determine whether post-intervention differences are attributable to the intervention effect or preexisting group differences. To control this, during the follow-up period all participants will be asked if they were included in any training regarding breastfeeding, those who will respond yes will be excluded from the follow-up assessment.
